# Novel fenoterol derivatives as suppressors of ERK1/2 phosphorylation in melanoma

**DOI:** 10.1007/s00210-026-05564-7

**Published:** 2026-06-20

**Authors:** Jakub Wójcik, Sylwia Wnorowska, Weronika Miziewicz, Giulia Tassinari, Gabriela Misiurek, Robert Kostecki, Agata Małek, Anna Boguszewska-Czubara, Irving W. Wainer, Artur Wnorowski

**Affiliations:** 1 Department of Biopharmacy, Medical University of Lublin, Lublin, Poland; 2 Doctoral Schoolof the Medical University of Lublin, Lublin, Poland; 3 Department of Medical Chemistry, Medical University of Lublin, Lublin, Poland; 4 Laboratory of Clinical Investigation, National Institute on Aging, National Institutes of Health, Baltimore, MD USA; 5 PAZ Pharma, Mullica Hill, Harrison, NJ USA

**Keywords:** Exchange protein directly activated by cAMP, Raf/MEK/ERK pathway, Naphthylfenoterol derivatives, Beta adrenergic receptor signaling, Stereochemistry, Pharmacological modulation of ERK signaling

## Abstract

Melanoma is a highly aggressive cancer with limited therapeutic options. In melanoma cell lines, β_2_AR activation leads to the suppression of pro-proliferatory MAPK/ERK signaling. This suggests that fenoterol, a potent β_2_AR agonist, can be useful in the treatment of melanoma. Here, we investigated the structure–activity relationship (SAR) of fenoterol derivatives for β_2_AR-depedent ERK suppression. We used the UACC-647 human-derived melanoma cell line to screen a panel of chiral compounds based on the fenoterol scaffold. The levels of phosphoactive ERK1/2 were quantified by immunoblotting. Anti-tumorigenic activity of the drugs was assessed using MTS assay and zebrafish xenograft model. (*R*,*R*′) stereoisomers of fenoterol derivatives exhibited the highest suppression of ERK phosphorylation, followed by (*R*,*S*′) and (*S*,*R*′) isomers, with (*S*,*S*′) isomers being the least active, mirroring trends observed in β_2_AR binding and cAMP accumulation. SAR analysis revealed that modifications to the α′ alkyl chain and the 4′-hydroxy moieties significantly affected ERK-inhibitory activity of fenoterol derivatives. (*R*,*R*′)-4′-hydroxy-1-naphthylfenoterol [(*R*,*R*′)-HNF] was the most active inhibitor of ERK activation with an IC_50_ of 0.03 nM. However, it showed minimal anti-proliferative activity in the zebrafish xenograft model. In contrast, (*R*,*R*′)-4′-methoxy-1-naphthylfenoterol [(*R*,*R*′)-MNF], a weaker ERK inhibitor (IC_50_ = 0.17 nM), significantly reduced cell viability in vitro (IC_50_ = 14.07 µM) and tumor growth in vivo. SAR for fenoterol-mediated ERK inhibition in melanoma was defined, highlighting the importance of the (*R*,*R*′) stereochemistry. Crucially, potent ERK inhibition does not directly predict anti-tumorigenic activity, as shown by (*R*,*R*′)-HNF. The pronounced anti-proliferatory effect of (*R*,*R*′)-MNF, despite weaker ERK inhibition, implies additional mechanisms beyond ERK modulation.

**Supplementary Information:**

The online version contains supplementary material available at https://doi.org/10.1007/s00210-026-05564-7.

## Introduction

Melanoma is one of the most aggressive cancers with poor prognosis and high rates of drug resistance (Soumoy et al. 2020). As the incidence rates continue to rise, it poses a great global health burden. Projections for 2025 estimate 104,960 new melanoma cases and 8,430 melanoma-associated deaths in the USA alone (Siegel et al. 2025), with similar trends observed in the European Union, anticipating 103,952 new cases and 17,258 deaths (European Union 2025).

β_2_-adrenergic receptor (β_2_AR) signaling has been implicated in the development and progression of cancer (Ogawa et al. 2020), including modulation of cell proliferation, apoptosis, and motility (Silva et al. 2024). β_2_AR belongs to the G-protein coupled receptor (GPCR) family, a large group of transmembrane receptors known to transduce extracellular signals into intracellular responses. GPCRs signaling influences the mitogen-activated protein kinase (MAPK) signal transduction pathway (Kwon et al. 2022). MAPKs are key regulators of melanoma development, and their dysregulation contributes to therapeutic resistance (Jagirdar et al. 2024;  Stauffer et al. 2024; Wu et al. 2023).

β_2_AR activation leads to G-protein-mediated modulation of cyclic AMP (cAMP) and triggers β-arrestin-dependent scaffolding that activates the Raf/MEK/ERK cascade  (Kahsai et al. 2023). The cAMP-dependent signaling further modulates the MAPK pathway (Wnorowski et al. 2022) primarily through two main effectors: protein kinase A (PKA) (Hoy et al. 2020) and the exchange protein directly activated by cAMP (EPAC) (Krishnan et al. 2022).

Both PKA and EPAC, the primary intracellular receptors for cAMP, exert complex, context dependent, and sometimes opposing effects on MAPK signaling. EPAC activation has been shown to induce ERK1/2 phosphorylation through pathways involving Rap1/BRAF/MEK (Keyes et al. 2020) and Rac1/p21/PAK/MEK (Uribe-Alvarez et al. 2021). Conversely, PKA is considered mostly inhibitory towards MAPK signaling; for instance, it can phosphorylate Src to induce Rap1-dependent inhibition of the Raf1/MEK/ERK axis (Schmitt and Stork 2002). However, the interplay between PKA and MAPK signaling is nuanced. PKA can also exhibit MAPK-enhancing properties through activation of GPCR kinases (GRKs) (Chrispell et al. 2022), which promote β-arrestin binding followed by scaffolding and activation of the RAF/MEK/ERK pathway, mirroring EPAC’s effects in some cellular contexts (Janetzko et al. 2022). Also, PKA forms a negative feedback loop on cAMP production by inhibiting adenylyl cyclase (AC) and enhancing cAMP hydrolysis by phosphodiesterases (PDEs) (Hoy et al. 2020).

**Fig. 1:**
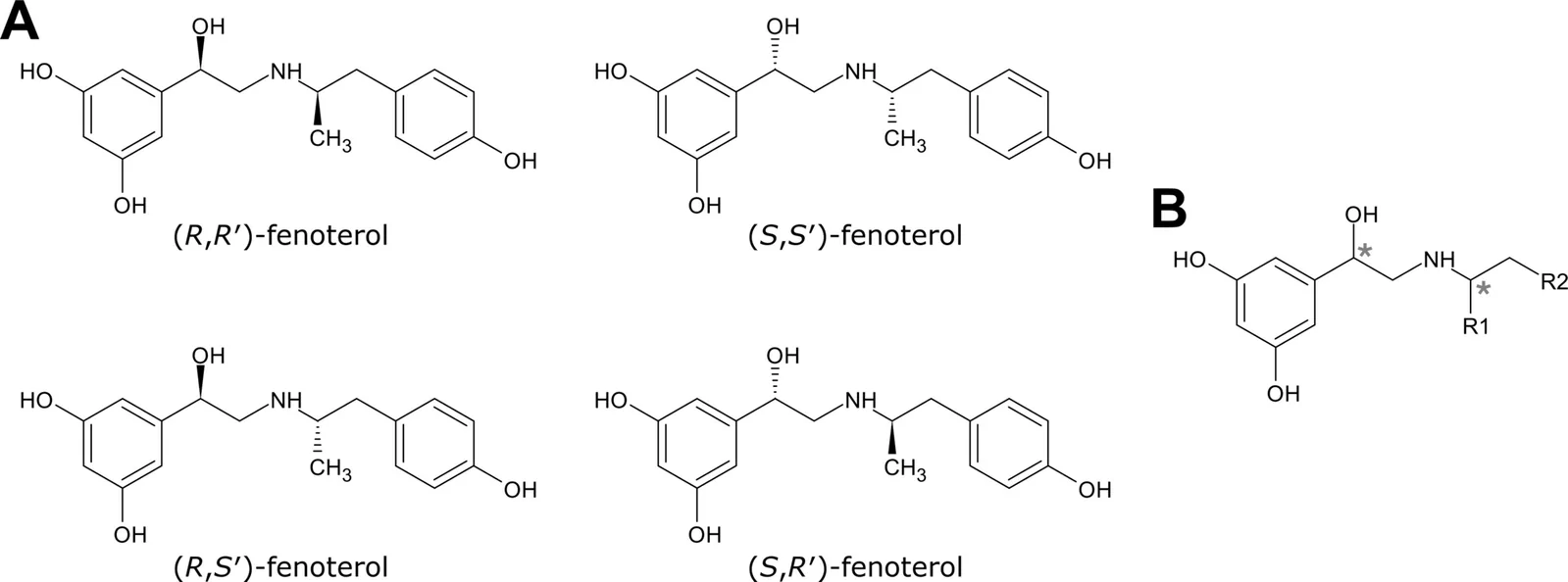
Structure of the compounds of interest.** A** Structure of the stereoisomers of fenoterol, the scaffold for the compounds investigated in this study. The presence of two stereocenters implicates that four different stereoisomers of the fenoterol exist, namely (*R*,*R*′), (*R*,*S*′), (*S*,*R*′), and (*S*,*S*′)-fenoterol. Marketed fenoterol is a racemic mixture of (*R*,*R*′) and (*S*,*S*′), with (*R*,*R*′)-fenoterol being responsible for the drug’s activity. **B** General structure of the fenoterol derivatives investigated in this study. Chiral carbon atoms are marked with red asterisks. R1 and R2 moieties are listed in Table 1

To probe the activity of β_2_AR in this context, we utilized fenoterol, a potent agonist originally developed for bronchodilation (Svedmyr 1985). Due to the presence of two chiral centers in its structure (Fig. 1), fenoterol exists as four stereoisomers: (*R*,*R*′), (*R*,*S*′), (*S*,*R*′), and (*S*,*S*′). Previous structure–activity relationship (SAR) studies have highlighted the critical role of stereochemistry in modulating the pharmacological profile of fenoterol derivatives. Jozwiak et al. (2007) and Plazinska et al. (2014) demonstrated that the stereochemical configuration of fenoterol derivatives, collectively referred to as *fenoterols*, profoundly influences their binding affinity and interactions within the β_2_AR binding pocket, suggesting that individual stereoisomers will have distinct effects on downstream signaling pathways.

The therapeutic potential of fenoterols as anti-cancer agents is supported by evidence across multiple malignancies. For instance, treatment with β_2_AR agonists, including fenoterols, markedly delayed proliferation and invasiveness of glioma cells (Wnorowski et al. 2017), while the fenoterol derivative 4′-methoxy-1-naphthylfenoterol (MNF) was shown to reprogram the metabolism of pancreatic cancer cells (Wnorowski et al. 2022). Our previous work demonstrated that (*R*,*R*′)-MNF potently inhibits ERK1/2 phosphorylation in melanoma cells in vitro via the activation of β_2_AR and downstream cAMP accumulation (Wnorowski et al. 2015). However, the key structural features of the fenoterol scaffold governing this inhibitory effect remain incompletely defined.

To elucidate the SAR for MAPK modulation by fenoterols, the current study expands this investigation to a comprehensive panel of derivatives and their corresponding stereoisomers with a focus on β_2_AR-mediated ERK1/2 inhibition in melanoma. We hypothesized that specific modifications to the fenoterol scaffold would significantly alter the potency of ERK inhibition and that the (*R*,*R*′) stereoisomer would consistently be the most active configuration. Using the β_2_AR-positive UACC-647 human-derived melanoma cell line, we screened a panel of fenoterol analogues to define these relationships. Finally, we sought to determine if the potency of ERK1/2 inhibition directly translates to anti-proliferative activity in vitro and in a zebrafish xenograft model, thereby testing the therapeutic relevance of this specific signaling node.

## Materials and methods

### Compounds of interest

Fenoterol (Fig. 1A) derivatives were synthesized as described previously by Jozwiak et al. (2007). The overall structure of the fenoterol scaffold is presented in Fig. 1B and the full list of studied compounds is presented in Table 1. All compounds were dissolved in DMSO (Merck, Germany, Darmstadt #D8418) to obtain stock solutions.

**Table 1:**
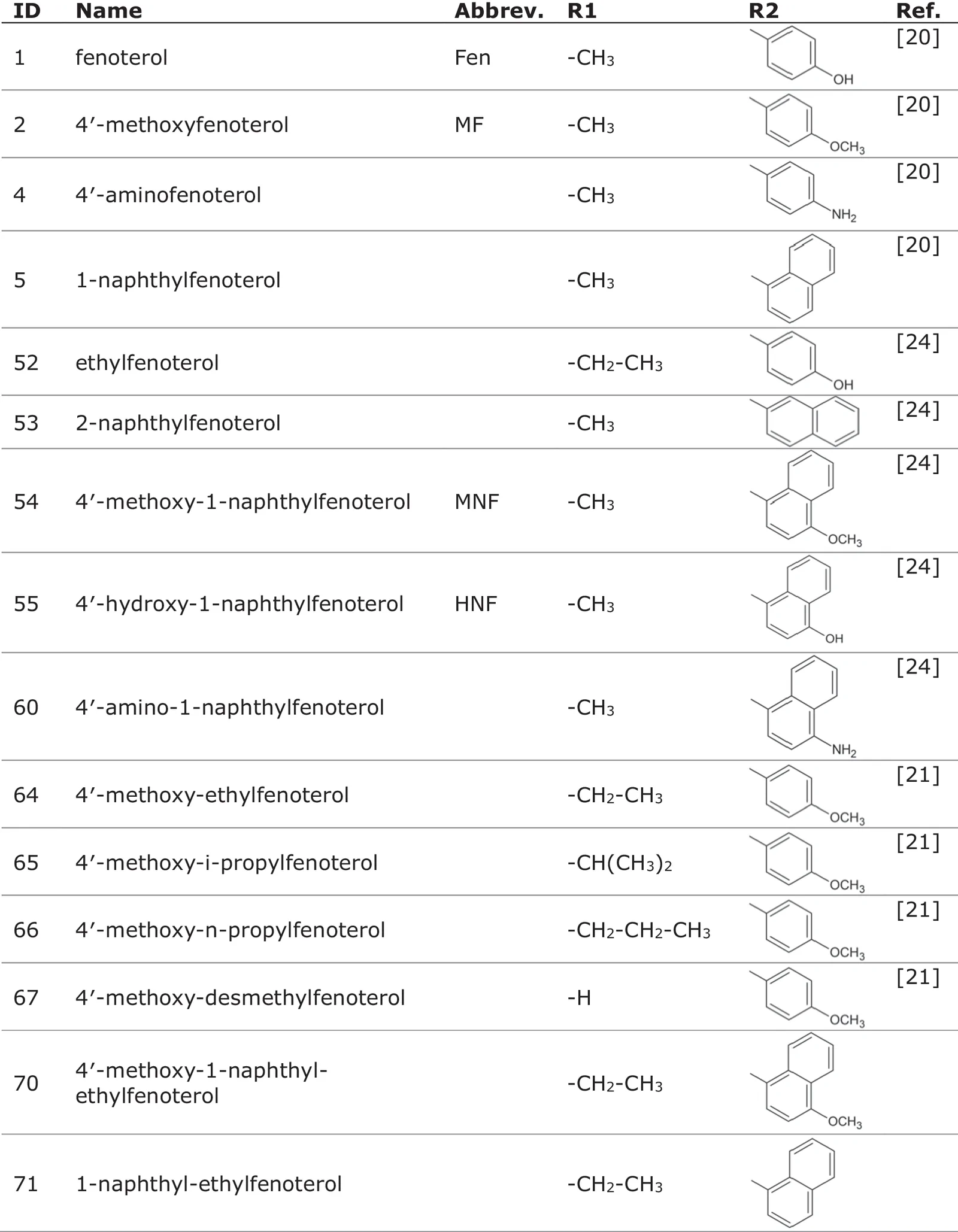
Names of compounds and residue structures

### Cell culture

UACC-647 (RRID:CVCL_4049) cell line was a generous gift from Michel Bernier (National Institute on Aging, National Institutes of Health, Baltimore, MD, USA). Colo-679 (RRID:CVCL_1130, #ACC 264), SK-MEL-30 (RRID:CVCL_0039, #ACC-151), SW480 (RRID:CVCL_0546, #ACC-313), and DU4475 (RRID:CVCL_1183, #ACC-427) cells were obtained from Leibniz Institute DSMZ-German Collection of Microorganisms and Cell Cultures GmbH, Braunschweig, Germany. U-87MG ATCC (RRID:CVCL_0022, #HTB-14) was obtained from ATCC, Manassas, VA, USA. UACC-647, Colo-679, SW480 and DU4475 cells were maintained in RPMI-1640 (Corning, Manassas, VA, USA, #10–104-CV), SK-MEL-30 in DMEM (Corning, #10–013-CV), and U-87MG ATCC in EMEM (Corning, #10–009-CV). All culture media were supplemented with 10 U/mL penicillin and 50 µg/mL streptomycin (Thermo Fisher Scientific (TFS), Waltham, MA, USA, #15,070,063) and 10% fetal bovine serum (Corning, #35–079-CV). The cell lines were maintained at 37 °C in a humidified atmosphere of 5% CO_2_.

### Quantitative polymerase chain reaction

The expression levels of adrenoceptor beta 2 (*ADRB2*) and G protein-coupled receptor 55 (*GPR55*) genes were measured by quantitative polymerase chain reaction (qPCR). β-actin (*ACTB*) housekeeping gene served as a reference. For this purpose, samples of 2 × 10^6^ cells were collected, snap-frozen in liquid nitrogen, and stored at − 80 °C. RNA was isolated using E.Z.N.A. HP Total RNA Kit (#R6812-01) according to the manufacturer’s protocol with the additional use of disruptor tubes (#AC1733-50) from Omega Bio-tek (Norcross, GA, USA).

Reverse transcription was performed using the SuperScript IV VILO Master Mix (TFS, #11,766,050), and the concentration of the obtained cDNA was normalized to 10 ng/µL for all samples. Next, qPCR was performed using SG qPCR Master Mix (EURx, Gdansk, Poland, #E0401) according to the manufacturer’s protocol with the addition of 2.5 µM primers for *ADRB2* (forward: AGGCCTTACCTCCTTCTTGC, reverse: GATCACCAGGGGAACGTAGA), *GPR55* (forward: CTGCCTTGGTTCCACCATA, reverse: CCAGGATGCAGGTGAGTAAGA), or *ACTB* (forward: AGAGCTACGAGCTGCCTGAC, reverse: AGCACTGTGTTGGCGTACAG). The qPCR reaction was performed using the LightCycler 480 Real-Time PCR System (Roche Diagnostics, Basel, Switzerland). Fluorescence was excited at 465 nm and measured at 510 nm.

Initial denaturation for 10 min in 95 °C was followed by 45 cycles of 95 °C for 10 s, 60 °C for 10 s and 72 °C for 20 s. After the reaction, high-resolution melting was performed from 60 to 95 °C at a ramp rate of 0.03 °C/s. The relative fold expression change was calculated using the ${2}^{{-\Delta \Delta C}_{T}}$ method, with *ACTB* serving as the housekeeping gene for normalization (Livak et al. 2001).

### Cell treatment

To assess the activity of fenoterol analogs on ERK phosphorylation, the UACC-647 cells were seeded in 6-well plates at 0.3 × 10^6^ cells per well and grown for 48 h at 37 °C. The medium was replaced with serum-free medium and the cells were starved for 3 h. Serum-starved cells were treated with different concentrations of fenoterol analogs (Table 1). Alternatively, the cells were exposed to the following EPAC1 inhibitors acting as downstream signaling modulators: ESI-09 (#4773) and CE3F4 (#4793), purchased from Tocris Bioscience. DMSO (0.1%) served as vehicle control. After 20 min of exposure, the cells were lysed, and cell lysates were analyzed by western blot.

### Western blotting

Protein level of GPR55 in all cell lines was determined by western blot. In UACC-647 cells treated with fenoterol derivatives, the levels of total and phosphorylated ERK1/2 were assayed.

Cells were lysed in 1 × Cell Lysis Buffer (Cell Signaling Technology (CST), Danvers, MA, USA, #9803) supplemented with phenylmethanesulfonyl fluoride (1 mM, Merck, #93,482-50ML-F) and 1 × Protease and Phosphatase Inhibitor Cocktail (TFS, #78,442). The protein level in the obtained lysates was determined using bicinchoninic acid assay (TFS, #23,227) with bovine serum albumin (TFS, #23,209) serving as a standard. Proteins were diluted using 6 × Laemmli SDS Sample Buffer and loaded at 10 µg/well on 4–12% precast gels (TFS, #NW04127BOX) for separation by polyacrylamide gel electrophoresis with 1 × MES-SDS (TFS, #B000202) as a running buffer. The separation was carried out at 90 V for 10 min and then at 120 V for an additional 60 min. Next, the proteins were transferred onto polyvinylidene fluoride membrane (TFS, #IB24001) by electroblotting using the iBlot2 system (TFS, #IB21001) programmed to conduct the following transfer steps: 20 V for 1 min, 23 V for 4 min, and 25 V for 2 min.

Subsequent membrane preparation involved blocking in 3% non-fat milk (Merck, #1.15363) in 1 × Tris-buffered saline with 0.1% Tween 20 (TBST) for 1 h, single rinsing with 1 × TBST, and overnight incubation at 4 °C with the primary antibodies against either GPR55 (NOVUS biologicals, #NB110-55498; TFS, #720,285; Abcam, #ab203663), total-ERK1/2 (CST, #4695) or phosphorylated ERK1/2 (Thr202/Tyr204, CST, #4370) used at 1:10,000 dilution in TBST with 3% BSA. The next day, the membrane was washed with 1 × TBST (two times for 4 min and two times for 2 min) and incubated with secondary goat anti-rabbit IgG antibodies conjugated with horseradish peroxidase (CST, #7074P2). The secondaries were diluted 1:10,000 in 1 × TBST with 3% non-fat milk. Before detection, the membrane was washed in 1 × TBST and dried from excessive moisture using disposable wipes. The detection of immunoreactive bands was performed by chemiluminescence using Westar Supernova ECL reagent (Cyanagen, Bologna, Italy, #XLS3). Images were acquired using Azure c400 (Azure Biosystems Inc., Dublin, CA, USA) until signal saturation. The optical density of each band was quantified on sub-saturated images using ImageJ 1.54f software (National Institutes of Health, Bethesda, MD, USA).

TBST was prepared using Tris hydrochloride (#T5941), Tris Base (#T6066), and NaCl (#793,566) from Merck as well as Tween 20 from SERVA Electrophoresis, Heidelberg, Germany (#39,796).

### Cell viability assay

Viability assay was performed on UACC-647 cells using the colorimetric MTS assay. The cells were seeded at 5000 cells/well in 96-well plates (NEST Biotechnology, Wuxi, Jiangsu, China, #701,001) and allowed to attach for 24 h. Then they were exposed to a gradient of (*R*,*R*′)-MNF or (*R*,*R*′)-HNF for 72 h. DMSO (0.1%) served as a vehicle control. MTS (Promega Corporation, Madison, WI, USA, #G111) and phenazine methosulfate (Merck, #P9625) in PBS (Corning, #21–031-CV) solution were added. After 3 h of incubation, the absorbance at 490 nm was measured spectrophotometrically using a BioTek Synergy H1 Multimode Reader (Agilent, Santa Clara, CA, USA).

### Xenograft experiment

Zebrafish embryos of the AB line (Centre of Experimental Medicine, Medical University of Lublin, Poland) were injected at 48 h post fertilization (hpf) with UACC-647 cells labelled with Vybrant DiI Cell-Labeling Solution (TFS, #V22885). Embryos bearing xenografts were imaged immediately after cancer cell implantation using a Zeiss V8 stereo microscope from ZEISS AG, Oberkochen, Germany (0-h time point). Then, the animals were exposed to (*R*,*R*′)-MNF and (*R*,*R*′)-HNF in E3 medium at the concentrations of 10, 25, and 50 µM. After 72 h of incubation, the animals were imaged again, and all obtained images were analyzed using ImageJ version 1.54f. Calculated integrated density values originating from DiI stain were normalized to vehicle-treated embryos at the 0-h time point.

### Statistical analysis

GraphPad Prism 10.4.0 (GraphPad Software, La Jolla, CA, USA) was used in calculating IC_50_ values for substance effect on relative dephosphorylation of ERK1/2 and cell viability using a four-parameter dose–response curve model. Pearson’s correlation coefficient was calculated using R version 4.3.3 (R Core Team 2004). For visualization, the data is represented as mean and 95% confidence interval (CI) standard deviation (SD) or standard error of the mean (SEM).

The effect of investigated substances on in vivo tumor growth in the xenograft experiment was analyzed using Data Analysis with Bootstrapped ESTimation (DABEST) package (Ho et al. 2019). Effect size was defined as the mean difference in mean fluorescence intensity of labelled cancer cells between the initial measurement and the 72-h end point. The data is represented in a Cumming plot as effect sizes and 95% CIs.

## Results

### Expression of β_2_AR and GPR55 in UACC-647 cells

**Fig. 2:**
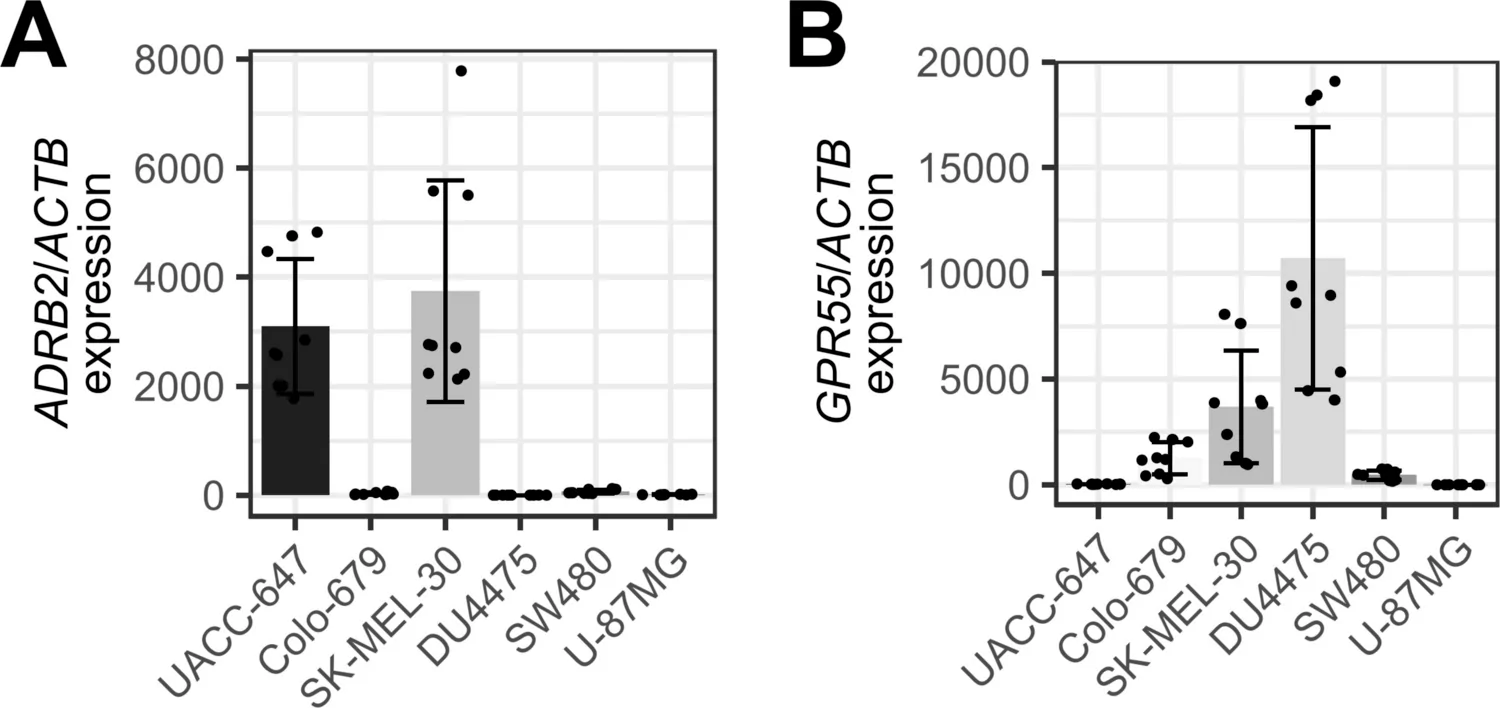
Expression of receptor coding genes. Relative fold expression change of adrenoceptor beta 2 (*ADRB2*) and G protein-coupled receptor 55 (*GPR55*) genes relative to β-actin (*ACTB*) housekeeping gene was measured by qPCR in melanoma (UACC-647, Colo-679 and SK-MEL-30) and other cancer cell lines of different origin (DU4475, SW480 and U-87MG). The data is represented as mean ± SD and individual values. The data originate from 3 independent biological replicates conducted in triplicates

Fenoterol derivatives were originally developed as selective β_2_AR agonists (Jozwiak et al. 2007; Plazinska et al. 2014; Jozwiak et al. 2010). However, some of these derivatives, including (*R,R*′) and (*R,S*′) stereoisomers of MNF, are also inhibitors of the G protein-coupled receptor GPR55 (Wnorowski et al. 2022; Paul et al. 2014). In the current study, we initially determined the expression of both receptors by qPCR in a panel of human melanoma and non-melanoma cell lines (Fig. 2).

UACC-647 melanoma cell line demonstrated a relatively high level of mRNA encoding β_2_AR (Fig. 2A), consistent with our previous immunoblotting and radioligand binding data (Wnorowski et al. 2015). Negligible *GPR55* expression was detected in UACC-647, especially in contrast to SK-MEL-30 and DU4475 cells characterized by abundant GPR55 (Fig. 2B). We sought to confirm qPCR results regarding GPR55 via western blotting. However, the specificity of commercially available anti-GPR55 antibodies was insufficient to obtain credible information about the protein expression of this receptor (data not shown).

As UACC-647 cell line exhibited high expression of *ADRB2* and not *GPR55*, it was chosen for use in the evaluation of specific β_2_AR-dependent activity of fenoterol derivatives.

### (*R*,*R*′) stereochemistry of fenoterols is optimal for ERK inhibition

**Fig. 3:**
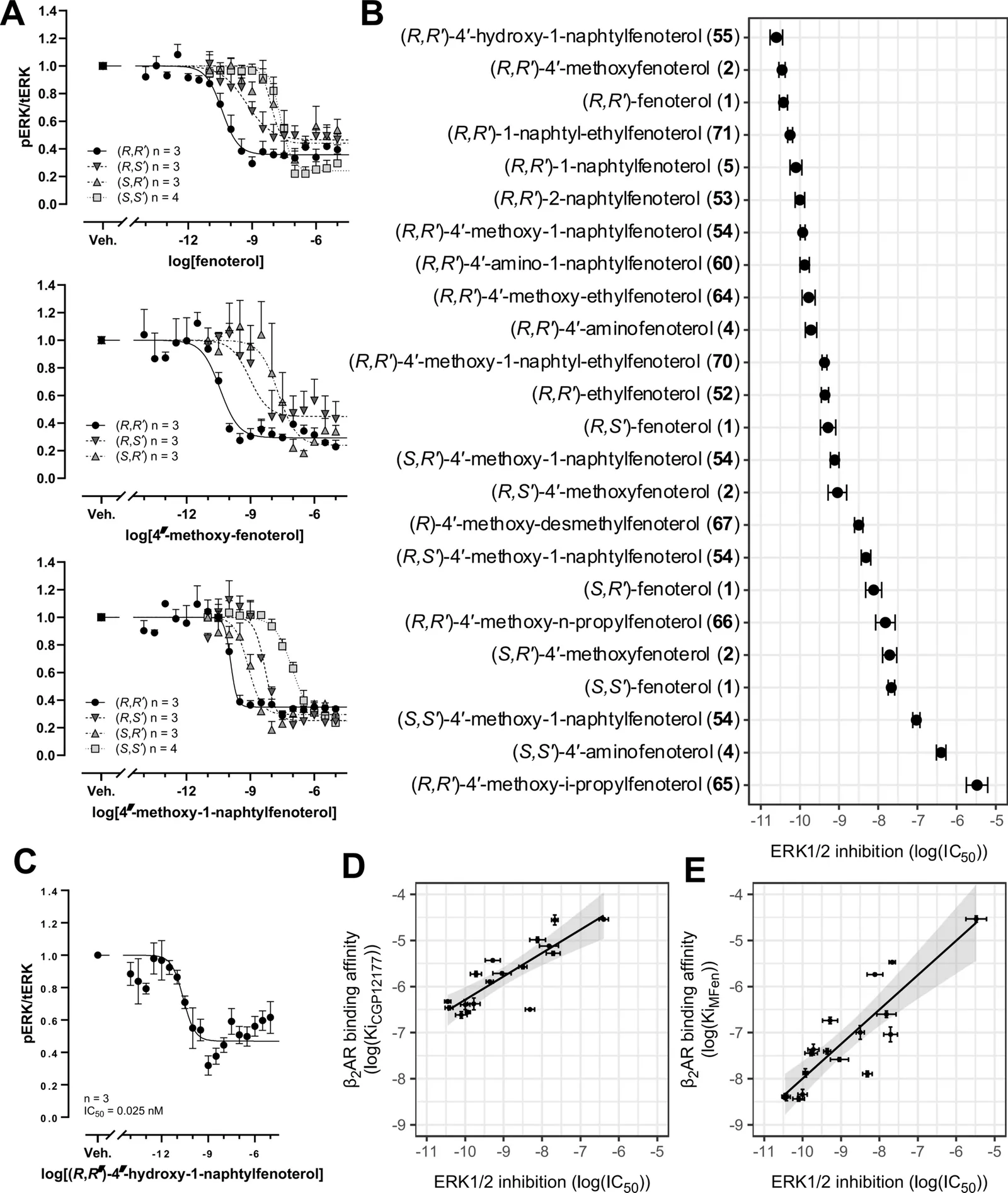
IC_50_values of ERK phosphorylation inhibition upon treatment with compounds of interest. Treatment with fenoterol derivatives (**A**) induces a reduction in ERK1/2 phosphorylation dependent of compound stereochemistry (**B**) and structure (**C**) in UACC-647 cells. Representative western blots are shown in Supplementary Figure S3. The number of biological replicates (*n*) is indicated on the dose–response graphs. ERK1/2 phosphorylation inhibition in UACC-647 cells correlates with β_2_AR binding affinity [data derived from Jozwiak et al. (2007), Jozwiak et al. (2010), and Plazinska et al. (2014) (**D**) and from Toll et al.(2012) and Plazinska et al. (2014) (**E**)]. The IC_50_ values were calculated using a four-parameter logistic dose response curve model using GraphPad Prism. Distinct shapes correspond to stereoisomers. Molar concentrations were log-transformed; data are presented as log(IC_50_) ± 95% CI for ERK1/2 phosphorylation inhibition, and as K_i_ or log(IC_50_) ± SEM

Fenoterol and its derivatives possess two chiral centers resulting in four possible stereoisomers (Fig. 1A). To determine the optimal stereochemical configuration for ERK1/2 inhibition, we initially compared the activity of the stereoisomers of fenoterol, 4′-methoxyfenoterol (MF) and MNF (Fig. 3A). For all tested compounds, the (*R*,*R*′) stereoisomer was consistently the most potent inhibitor of ERK1/2 phosphorylation (Fig. 3A, Supplementary Table S1). For example, the IC_50_ of (*R*,*R*′)-fenoterol was 0.038 nM, whereas the other fenoterol isomers were significantly less active, i.e., (*R*,*S*′) and (*S*,*R*′) stereoisomers exhibited an intermediate rate of inhibition yielding the IC_50_ of 0.521 nM and 7.638 nM, respectively, and (*S*,*S*′) isomer displayed the lowest activity with the IC_50_ of 21.577 nM. This activity pattern—(*R*,*R*′) > (*R*,*S*′) ≈ (*S*,*R*′) > (*S*,*S*′)—establishes the (*R*,*R*′) configuration as optimal for this signaling effect.

### SAR of fenoterols defines key moieties for ERK inhibition

Having established the importance of the (*R*,*R*′) configuration, we next investigated the SAR by comparing derivatives with modifications at the α′ alkyl chain (R1) and the phenyl ring (R2).

#### Modification of the α′ alkyl chain (R1)

Elongating the methyl group of (*R*,*R*′)-fenoterol to an ethyl group resulted in a more than ten-fold decrease in potency (Fig. 3B). A similar trend was observed when elongating the α′ chain of (*R*,*R*′)-MNF and (*R*,*R*′)-MF (IC_50_ increased 3.6-fold and ~ fivefold, respectively). Further elongation to a propyl group or introducing branching (i-propyl) dramatically reduced activity, with IC_50_ values increasing by orders of magnitude. Similarly, removing the alkyl chain at R1 markedly suppressed the inhibitory activity of desmethyl derivative (Fig. 3B, Supplementary Table S1). These data indicate that a methyl group at the α′ position is optimal for potent ERK1/2 inhibition.

#### Modification of the phenyl ring (R2)

To evaluate the influence of the R2 aromatic moiety, we first assessed the effects of modifying the 4′-hydroxyl group on the fenoterol phenyl ring. Replacing the 4′-hydroxyl moiety of (*R*,*R*′)-fenoterol with a methoxy group maintained high potency (IC_50_ = 0.035 nM vs. 0.038 nM for fenoterol), whereas substitution with an amino group [(*R*,*R*′)−4′-aminofenoterol] resulted in a fivefold reduction in ERK1/2 inhibitory activity (IC_50_ = 0.191 nM). This reduction in activity is consistent with previous binding studies and CoMFA models indicating that electronegative substituents at this position are preferred for optimal interaction with the β_2_AR (Jozwiak et al. 2007).

We next investigated the effects of substituents on the 1-naphthyl scaffold (Fig. 3B, Supplementary Table S1). The unsubstituted (*R*,*R*′)−1-naphthylfenoterol exhibited strong activity (IC_50_ = 0.079 nM). Introducing a 4′-hydroxyl group to this scaffold yielded (*R*,*R*′)-HNF, the most potent compound in this study (IC_50_ = 0.025 nM). However, unlike the phenyl series where the methoxy substitution preserved potency, converting the 4′-hydroxyl of the naphthyl scaffold to a methoxy [(*R*,*R*′)-MNF] or amino [(*R*,*R*′)-ANF] group decreased activity approximately fivefold (IC_50_ values of 0.118 nM and 0.132 nM, respectively). This suggests that while the extended aromatic system of the naphthyl ring enhances affinity through hydrophobic and π-π interactions (Jozwiak et al. 2007), the steric constraints imposed by this scaffold make the hydrogen-bonding capability of the 4′-substituent even more critical. The superior potency of the hydroxyl group implies that its capacity to act as both a hydrogen bond donor and acceptor provides the most favorable electrostatic fit within the agonist-stabilized receptor conformation.

Finally, we confirmed the specific structural modifications affecting ERK1/2 phosphorylation potency correlate strongly with previously reported pharmacological parameters of the fenoterols. The ERK inhibition IC_50_ values showed a strong positive correlation with K_i_ values from both [^3^H]CGP12177 (Pearson’s *r* = 0.84) and [^3^H](*R*,*R*′)-MF (Pearson’s *r* = 0.88) binding assays (Fig. 3D–E), indicating the effect is β_2_AR-mediated. Furthermore, to verify that the observed ERK suppression is mechanically linked to the canonical β_2_AR-G_s_ signaling axis, we correlated our data with potencies for G_s_α GTPase activation and AC stimulation reported for stereoisomers of fenoterol, MF, and MNF (Reinartz et al. 2015). We observed a strong positive correlation between ERK1/2 inhibition and both G_s_α GTPase activity (*r* = 0.96) and AC activation (*r* = 0,82) (Supplementary Fig. S1A–B). Collectively, these correlations demonstrate that the structure–activity relationship governing ERK1/2 inhibition in UACC-647 cells mirrors the SAR established for receptor binding and canonical G_s_ protein activation.

#### EPAC is involved in ERK1/2 inhibition in UACC-647 cells

**Fig. 4:**
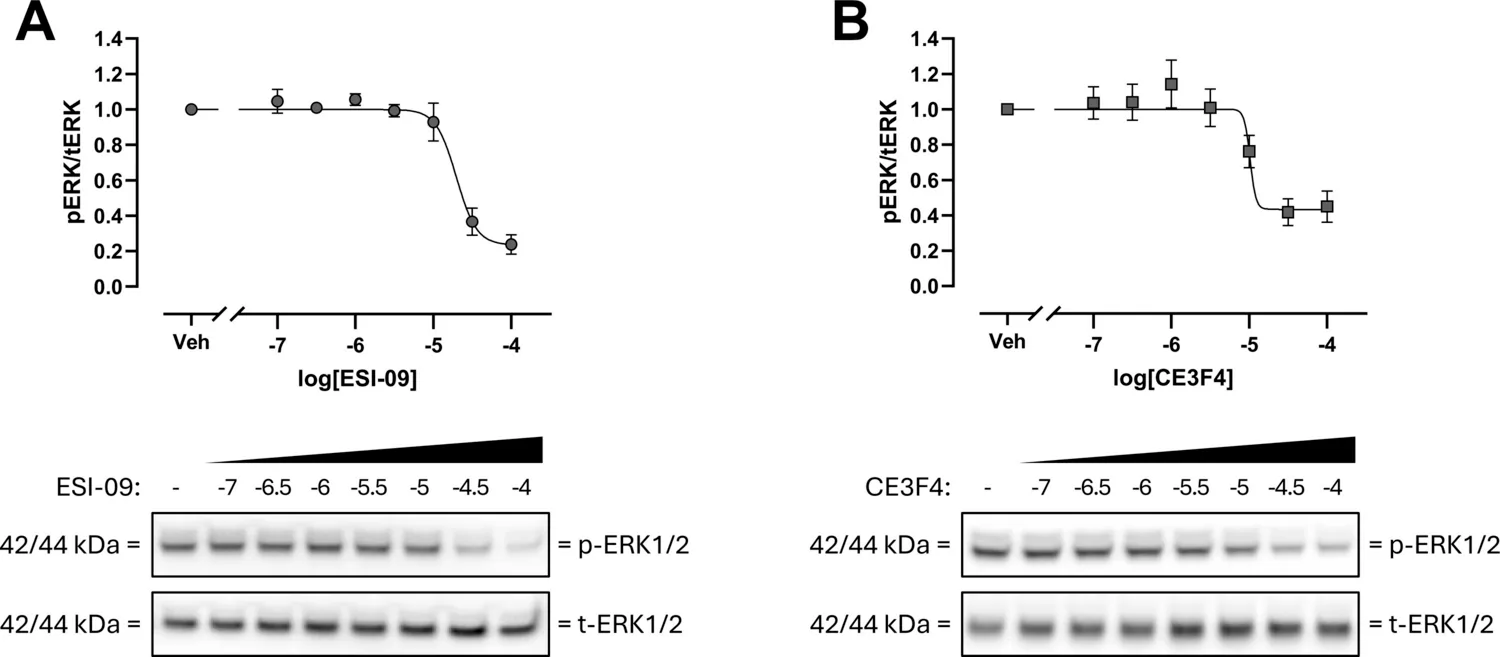
Pharmacological inhibition of EPAC leads to the suppression of ERK1/2 phosphorylation in UACC-647 cells. Cells were incubated in serum-free medium for 3 h and then exposed for 20 min to increasing concentrations of ESI-09 (**A**) or CE3F4 (**B**). DMSO (0.1%) served as a vehicle control. Total and phosphorylated forms of ERK1/2 were visualized via western blot or dot blot and the relative intensity of each band was measured densitometrically. The ratio between phosphoactive and total ERK1/2 is presented as mean ± SEM (*n* = 8). The IC_50_ values were calculated using curve fitting to a four-parameter logistic equation. Data originate from five independent experiments. Uncropped blot images are provided in the Supplementary Fig. S2

Our previous study revealed that PKA pathway is crucial for β_2_AR-dependant ERK1/2 inhibition (Wnorowski et al. 2015; Wnorowski et al. 2017). As EPAC is another significant signal transducer downstream of cAMP, we utilized two distinct chemical inhibitors to evaluate the role of EPAC in ERK1/2 modulation in UACC-647 cells. Surprisingly, both inhibitors dose-dependently decreased ERK1/2 phosphorylation with IC_50_ values of 19.8 µM (Fig. 4A) and 10.3 µM (Fig. 4B), respectively. This result indicates that, in contrast to PKA, EPAC provides a constitutive tone that promotes ERK1/2 activation in these cells. Therefore, β_2_AR-mediated ERK1/2 suppression likely occurs through PKA-dependent inhibition that overrides this basal EPAC-driven activation.

### β_2_AR-dependent ERK1/2 inhibition does not directly translate into antiproliferative effect

**Fig. 5:**
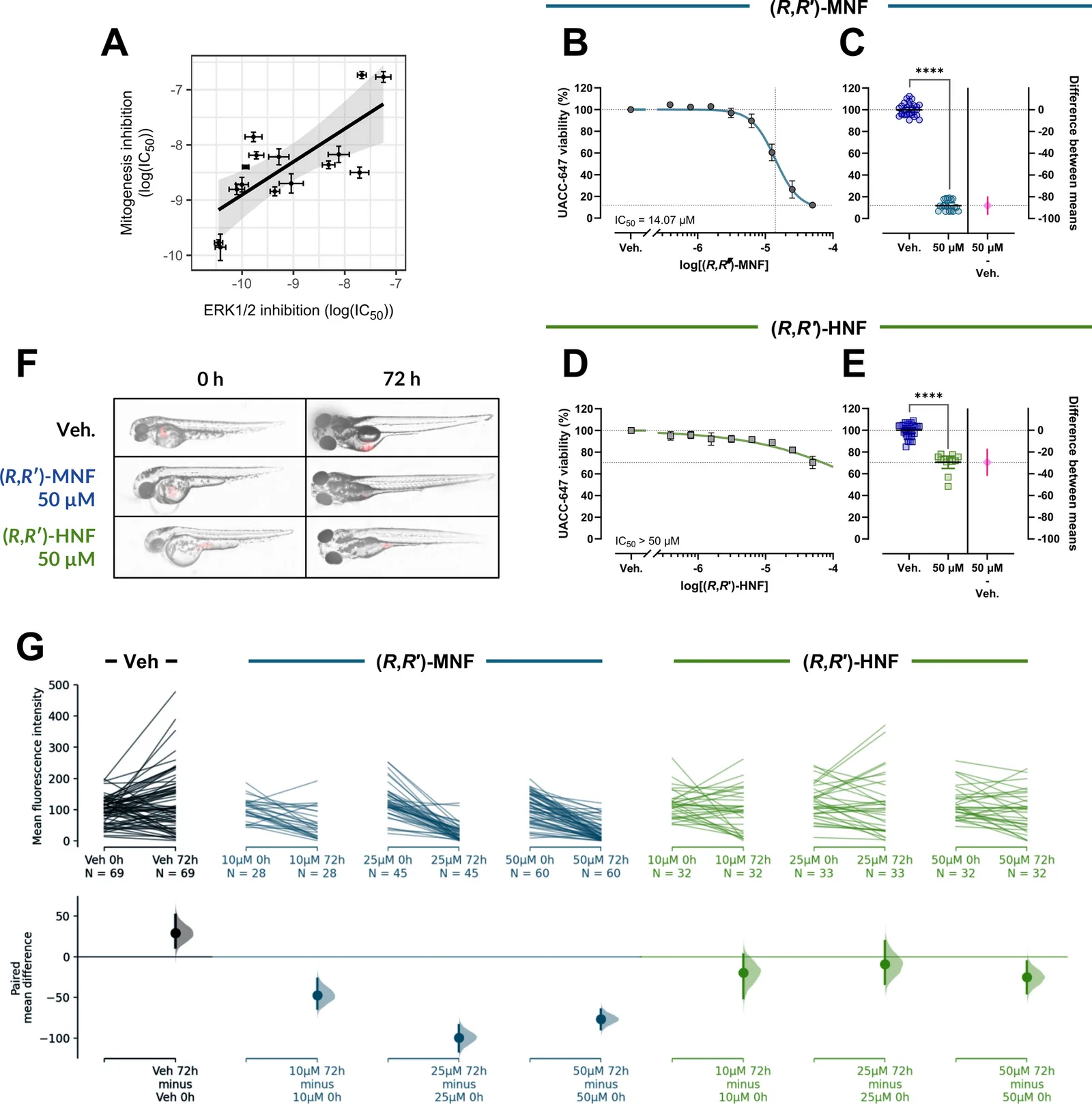
(*R*,*R*′)−4′-methoxy-1-naphthylfenoterol [(*R*,*R*′)-MNF], but not (*R*,*R*′)−4′-hydroxy-1-naphtylfenoterol [(*R*,*R*′)-HNF], inhibits the growth of UACC-647 in vitro and in vivo. **A** Correlation between the inhibitory activity of fenoterols towards ERK1/2 phosphorylation in UACC-647 cells (horizontal axis) and the capacity of the same compounds to suppress the mitogenesis in 1321N1 cells (vertical axis). (**B** and **C**) UACC-647 melanoma cells were exposed to a gradient of (*R*,*R*′)-MNF (**B** and **C**) or (*R*,*R*′)-HNF (**D** and **E**) for 72 h in three independent biological experiments. DMSO (0.1%) served as a vehicle control. Cell viability was assessed using MTS assay. Dose–response curves were generated by fitting control-normalized experimental data to four-parameter logistic equation. **B** The IC_50_ value for (*R*,*R*′)-MNF was equal to 14.07 µM (95% CI: 12.79 to 15.74 µM). **C** The mean difference between the normalized viability observed for the vehicle-treated cells (Veh.) and for the cells exposed to a 50 µM (*R*,*R*′)-MNF is shown in the Gardner-Altman estimation plot. Both groups are plotted on the left axis; the mean difference is plotted on the axis on the right. The mean difference is depicted using a diamond symbol; the 95% confidence interval is indicated by the ends of the vertical error bar. The unpaired mean difference between Veh. and 50 µM (*R*,*R*′)-MNF was − 88.1% (95.0%CI: − 91.1 to − 85.0%). **D** It was not possible to calculate IC_50_ value for (*R*,*R*′)-HNF, as this compound elicited only minor suppression of cell fitness. **E** The unpaired mean difference in cell viability between DMSO (Veh.) and 50 µM (*R*,*R*′)-HNF was − 29.5% (95.0% CI: − 36.6 to − 25.4%). **F** Zebrafish embryos were injected at 48 h post fertilization (hpf) with UACC-647 cells labelled with DiI membrane stain. Embryos bearing xenografts were imaged immediately after cancer cell implantation (0-h time point). Then, the animals were exposed to the compounds of interest for 72 h and imaged again. **G** Calculated integrated density values originating from DiI stain were normalized to vehicle-treated embryos at 0-h time point and plotted. The paired mean difference for 7 comparisons are shown in the above Cumming estimation plot. The vehicle-normalized data is plotted on the upper axes; each paired set of observations is connected by a line. On the lower axes, each paired mean difference is plotted as a bootstrap sampling distribution. Mean differences are depicted as dots; 95% confidence intervals are indicated by the ends of the vertical error bars. In vivo experiments were carried out in three independent fish cohorts for each compound

Given that MAPK signaling is a key driver of melanoma progression (Savoia et al. 2019), we hypothesized that potent ERK1/2 inhibition by fenoterols would correlate with anti-proliferative activity. A preliminary analysis showed a moderate correlation (Pearson’s *r* = 0.74) between ERK1/2 inhibition in UACC-647 cells (this study) and mitogenesis suppression in β_2_AR-positive 1321N1 cells (previously published data, Toll et al. (2011)) (Fig. 5A), supporting the hypothesis.

To test this directly, we compared the cytotoxic effects of the most potent ERK inhibitor identified here, namely (*R*,*R*′)-HNF (Fig. 3B), along with another fenoterol derivative—(*R*,*R*′)-MNF, a previously established inhibitor of melanoma proliferation (Wnorowski et al. 2015). Despite being a ~ sixfold weaker ERK inhibitor, (*R*,*R*′)-MNF reduced UACC-647 viability in vitro with IC_50_ = 14.07 µM (Fig. 5B) and at the dose of 50 µM, it generated 88.1% drop in cellular viability (Fig. 5C). In stark contrast, the highly potent ERK inhibitor (*R*,*R*′)-HNF displayed only minimal cytotoxic effect (Fig. 5D) with viability decreased by only 29.5% at the same 50 µM dose (Fig. 5E).

This dissociation between ERK inhibition and anti-proliferative activity was confirmed in a zebrafish xenograft model. Control tumors grew by 29.4%, confirming the proliferative capacity of the grafted cells, while treatment with (*R*,*R*′)-MNF significantly inhibited their growth in vivo at all tested concentrations, with the maximal effect of 99.6% inhibition observed at a 25 µM dose (Fig. 5F–G). However, (*R*,*R*′)-HNF displayed a significantly lower effect on tumor growth, with the decrease reaching only 25.3% at a 50 µM dose (Fig. 5G). These results demonstrate that potent, acute inhibition of ERK1/2 phosphorylation by fenoterols is not a predictor of their anti-tumor efficacy.

## Discussion

In this study, we sought to define the structure–activity relationship for fenoterol-mediated suppression of ERK1/2 signaling in melanoma. Our screening of fenoterol analogues identified (*R*,*R*′)−4′-hydroxy-1-naphthylfenoterol [(*R*,*R*′)-HNF] as the most potent derivative for inhibiting ERK1/2 phosphorylation. We confirmed that the (*R*,*R*′) stereoisomers consistently exhibited the highest efficacy and that this activity correlated strongly with β_2_AR binding affinity. Intriguingly, we discovered that potent ERK1/2 inhibitory activity does not predict anti-proliferative effects in vitro or in vivo. These results provide a refined SAR for fenoterols in melanoma and, more importantly, highlight a critical disconnect between acute pathway modulation and cellular outcomes, emphasizing the complex relationship between β_2_AR signaling, ERK modulation, and anti-tumorigenic activity.

Our results confirm and extend previous findings on the critical role of stereochemistry in fenoterols pharmacology. The observed activity hierarchy for ERK1/2 inhibition—(*R*,*R*′) > (*R*,*S*′) ≈ (*S*,*R*′) > (*S*,*S*′)—precisely mirrors the pattern previously reported for cAMP accumulation in HEK297 cells overexpressing β_2_AR (Jozwiak et al. 2010) and for anti-mitogenic effects in 1231N1 astrocytoma cells (Toll et al. 2011). Recent investigations into the structure-bias relationships of fenoterol stereoisomers suggest that the (*R*,*R*′) configuration is critical not only for binding affinity but also for functional efficacy (Reinartz et al. 2015). Littmann et al. demonstrated that while (*R*,*R*′)-isomers are highly efficacious for both G_s_ activation and β-arrestin recruitment, deviation from this stereochemistry [e.g., in (*R*,*S*′)**-**isomers] leads to a drastic loss of β-arrestin coupling, effectively creating G_s_-biased ligands (Littmann et al. 2015). Thus, the (*R*,*R*′) configuration appears to be the eutomer required to fully engage the receptor’s orthosteric and exosite binding pockets (Proudman and Baker 2025) to trigger maximal downstream signaling. This consistency across different cell types and functional readouts strongly suggests that the initial stereoselective binding event at the β_2_AR is the primary determinant of downstream efficacy for this signaling axis. The strong correlation between our ERK inhibition data and published binding affinities (Jozwiak et al. 2007) further solidifies this conclusion. Similarly, our SAR analysis reinforces the established model of the fenoterol binding pocket: the superior activity of compounds with a methyl group at the R1 position and a hydroxyl (or naphthyl-hydroxyl) at the R2 position highlights the strict structural requirements for optimal receptor activation.

The current study, together with previous observations (Wnorowski et al. 2015), suggests opposing roles for PKA and EPAC in regulating ERK signaling in UACC-647 melanoma cells. While our previous work showed that PKA activation is inhibitory (Jozwiak et al. 2010), our current data reveal that pharmacological inhibition of EPAC also suppresses ERK1/2 phosphorylation, in accordance with other studies. This implies that, at baseline, EPAC provides a tonic, pro-proliferative signal that promotes ERK activation. Therefore, the net effect of β_2_AR agonism that translates into the potent ERK inhibition is not simply due to cAMP production, but likely results from a PKA-mediated inhibitory signal that is strong enough to override the ongoing EPAC-driven activation. This raises the possibility that β_2_AR activation generates spatially or functionally compartmentalized cAMP pools that preferentially engage PKA over EPAC, a mechanism of signal specification that has been described in other systems  (Rudokas et al. 2020; Zaccolo et al. 2021; Yadav and Zaccolo 2025).

It is also important to note that the β_2_AR classically exhibits dual coupling to both G_s_ and G_i_ proteins  (Daaka et al. 1997). While G_i_ coupling is frequently associated with the activation of the MAPK/ERK cascade (Koch et al. 1994), the net acute response (at 20 min) to β_2_AR activation in UACC-647 cells is a profound inhibition of ERK1/2. This phenomenon can be partially attributed to “system bias,” an effect where the specific stoichiometry of receptor transducers and downstream effectors in a given cellular setting dictates the dominant signaling output (Smith et al. 2018). It appears that the UACC-647 melanoma model is inherently wired to favor the G_s_/cAMP/PKA signaling axis. Because of this pronounced system bias, the robust G_s_-mediated inhibitory signal overrides both the basal EPAC tone and any potential G_i_-mediated activating signals. Indeed, we previously observed that even the balanced β_2_AR agonist isoproterenol potently suppresses ERK1/2 phosphorylation in UACC-647 cells (Wnorowski et al. 2015).

Given that ERK1/2 activation is a key driver of cell proliferation and MAPK inhibitors are clinically relevant in melanoma, we hypothesized that suppressive activity towards phosphoactive ERK1/2 observed in response to fenoterols will contribute to a reduction in UACC-647 viability in vitro and in vivo. Despite promising results on ERK1/2 inhibition, (*R*,*R*′)-HNF was ineffective as an anti-proliferative agent in cultured UACC-647 cells and in xenograft model in zebrafish. (*R*,*R*′)-MNF, a compound less potent in terms of phospho-ERK downregulation, demonstrated noticeable activity both in vitro and in vivo.

A crucial limitation of this study is that its design focused on the acute effect of fenoterol derivatives on MAPK signaling. A similar study on MAPK pathway modulation in melanoma showed that effects on cell viability do not always correlate with pathway activity (Krishnan et al. 2022). The dissociation between acute ERK inhibition and long-term anti-proliferative efficacy observed for (*R*,*R*′)-HNF and (*R*,*R*′)-MNF likely reflects the phenomenon of ligand-biased signaling rather than off-target effects. Although (*R*,*R*′)-MNF is a known antagonist of the pro-proliferative GPR55 receptor  (Wnorowski et al. 2022;  Paul et al. 2014), our data confirms that UACC-647 cells lack GPR55 expression (Fig. 2B), implying the observed cytotoxicity is driven primarily by the β_2_AR. The structural difference between these two ligands (4′-methoxy-1-naphthyl moiety in MNF versus the 4′-hydroxy-1-naphthyl in HNF) has been shown to fundamentally alter receptor coupling. Reinartz et al. (2015) and Littmann et al. (2015) reported that the bulky naphthyl tail creates steric hindrance that disproportionately impairs β-arrestin recruitment while preserving G_s_ signaling. We propose that (*R*,*R*′)-HNF, with its catechol-like 4′-hydroxyl group, acts as a balanced agonist that induces temporal switch from G_s_ to G_i_ coupling followed by robust β-arrestin recruitment, leading to rapid receptor desensitization and internalization. This temporal progression would render the receptor incapable of sustaining the G_s_/PKA inhibitory tone, restricting the ERK suppression to an acute, transient pulse. In contrast, (*R*,*R*′)-MNF, acting as a ligand with slight G_s_ preference over arrestin recruitment (Littmann et al. 2015; Reinartz et al. 2015), likely evades rapid desensitization. By bypassing the temporal switch to G_i_, (*R*,*R*′)-MNF maintains a sustained cAMP/PKA signaling output over time. This hypothesis is supported by our previous demonstration that (*R*,*R*′)-MNF elicits a prolonged suppression of ERK1/2 phosphorylation lasting at least 48 h in UACC-647 cells (Wnorowski et al. 2015). This would allow for sustained cAMP/PKA signaling necessary to drive the anti-proliferative phenotype observed in viability assays.

## Supplementary Information

Below is the link to the electronic supplementary material.

ESM 1 (PDF 2.35 MB)

## Data Availability

Data supporting the findings of this study are available within the paper and its Supplementary Information. Raw images can be obtained from the corresponding author.

## Abbreviations

AC: Adenylyl cyclase
*ACTB*: β-Actin
β_2_AR: β_2_-Adrenergic receptor
cAMP: Cyclic adenosine monophosphate
CI: Confidence interval
EPAC: Exchange protein directly activated by cAMP
GPCR: G-protein coupled receptor
GPR55: G-protein coupled receptor 55
GRK: GPCR kinase
HNF: 4′-Hydroxy-1-naphthylfenoterol
hpf: Hours post fertilization
MAPK: Mitogen-activated protein kinase
MF: 4′-Methoxyfenoterol
MNF: 4′-Methoxy-1-naphthylfenoterol
PDE: Phosphodiesterase
PKA: Protein kinase A
SD: Standard deviation
SEM: Standard error of the mean
TBST: Tris-buffered saline with 0.1% Tween 20
