# Supplementary material for: Novel fenoterol derivatives as suppressors of ERK1/2 phosphorylation in melanoma
Source: Naunyn Schmiedebergs Arch Pharmacol. 2026 Jun 20;399(12):19201–13. doi: 10.1007/s00210-026-05564-7 (PMC13593774; doi:10.1007/s00210-026-05564-7)
Supplement: Supplementary file 1 [file 210_2026_5564_MOESM1_ESM.pdf]

Supplementary Material

Novel fenoterol derivatives as suppressors of ERK1/2 phosphorylation in melanoma

Jakub Wójcik[1,2], Sylwia Wnorowska[3], Weronika Miziewicz[1,2], Giulia Tassinari[1], Gabriela Misiurek[1], Robert Kostecki[1], Agata Małek[2,3], Anna Boguszewska-Czubara[3], Irving W. Wainer[4,5], Artur Wnorowski[1]*

[1] Department of Biopharmacy, Medical University of Lublin, Lublin, Poland

[2] Doctoral School of the Medical University of Lublin, Lublin, Poland

[3] Department of Medical Chemistry, Medical University of Lublin, Lublin, Poland

[4] Laboratory of Clinical Investigation, National Institute on Aging, National Institutes of Health, Baltimore, MD, USA

[5] PAZ Pharma, Mullica Hill, NJ, USA

* The correspondence should be addressed to: Artur Wnorowski, email: artur.wnorowski@umlub.edu.pl

Table S1. Activities of fenoterol derivatives and their stereoisomers.

ID	Name and abbreviations	R1	R2	Stereo-chemistry	β_2AR binding affinity† $Ki_{CGP12177}$ (nM) ± SEM	β_2AR binding affinity† Ki_{MFen} (nM) ± SEM	cAMP stimulation‡ EC_{50} (nM) ± SEM	cAMP stimulation‡ Max. cAMP stimulation (%) ± SEM	Mitogenesis inhibition§ IC_{50} (nM) ± SEM	GTPase activation of β_2AR-$G_s\alpha$ fusion$ log(EC_{50}) ± SD	AC activation via $G_s\alpha$€ log(EC_{50}) ± SD	ERK1/2 inhibition‖ IC_{50} (nM) (95% CI)	ERK1/2 inhibition‖ Max. ERK1/2 inhibition (%) (95% CI)
1[a]	fenoterol	-CH_3	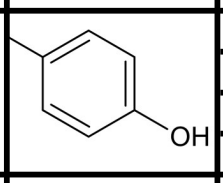	(*R*,*R'*)	345.15 ± 33.83[a]	4.00 ± 0.75[d]	0.30 ± 0.09[c]	123.30 ± 8.79[c]	0.14 ± 0.07[e]	-8.03 ± 0.06[g]	-8.01 ± 0.13[g]	0.038 (0.023 - 0.059)	64.19 (60.20 - 68.30)
				(*R*,*S'*)	3695.00 ± 245.79[a]	183.00 ± 30.00[d]	4.70 ± 0.50[c]	130.50 ± 30.33[c]	6.09 ± 1.93[e]	-7.08 ± 0.04[g]	-7.32 ± 0.13[g]	0.521 (0.216 - 1.321)	53.56 (48.00 - 60.10)
	Fen[b,d]			(*S*,*R'*)	10330.16 ± 1405.65[a]	1827.00 ± 117.00[d]	8.50 ± 4.16[c]	124.70 ± 5.16[c]	6.74 ± 2.18[e]	-6.30 ± 0.08[g]	-6.08 ± 0.09[g]	7.638 (2.323 - 15.382)	55.92 (47.99 - 64.09)
				(*S*,*S'*)	27749.95 ± 6816.14[a]	3370.00 ± 210.00[d]	580.20 ± 102.97[c]	126.30 ± 8.19[c]	184.27 ± 26.13[e]	-5.93 ± 0.06	-5.93 ± 0.10[g]	21.577 (14.388 - 31.189)	75.83 (69.74 - 82.20)
2[a]	4′-methoxyfenoterol	-CH_3	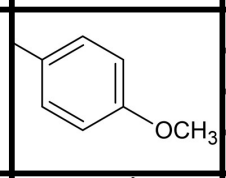	(*R*,*R'*)	473.78 ± 34.73[a]	4.09 ± 0.55[d]	0.30 ± 0.23[c]	135.70 ± 11.03[c]	0.17 ± 0.02[e]	-8.07 ± 0.12[g]	-8.94 ± 0.14[g]	0.035 (0.024 - 0.051)	69.26 (65.31 - 73.26)
	MF			(*R*,*S'*)	1929.83 ± 135.07[a]	26.10 ± 2.44[d]	2.00 ± 0.45[c]	120.10 ± 18.97[c]	2.01 ± 0.76[e]	-7.40 ± 0.09[g]	-7.45 ± 0.15[g]	0.908 (0.286 - 2.529)	55.22 (46.02 - 64.63)
	MFen[d]			(*S*,*R'*)	5268.93 ± 508.84[a]	91.30 ± 32.01[d]	7.20 ± 1.01[c]	129.00 ± 12.67[c]	3.16 ± 0.71[e]	-6.19 ± 0.14[g]	-6.51 ± 0.13[g]	19.498 (8.222 - 42.855)	73.58 (62.02 - 85.67)
	MethoxyFen[b,d]			(*S*,*S'*)	15880.54 ± 2722.55[a]	2870.00 ± 234.00[d]	33.20 ± 11.48[c]	156.40 ± 35.93[c]	337.20 ± 97.20[e]	-5.72 ± 0.13[g]	-5.90 ± 0.22[g]		
3[a]	phenylfenoterol	-CH_3	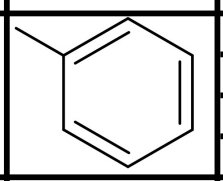	(*R*,*R'*)	2933.00 ± 238.00[a]	26.60 ± 1.49[d]							
				(*R*,*S'*)	7937.00 ± 561.00[a]								
	PhFen[b,d]			(*S*,*R'*)	23125.00 ± 2093.00[a]								
	PhenylFen[d]			(*S*,*S'*)	28600.00 ± 900.00[a]								
4[a]	4′-aminofenoterol	-CH_3	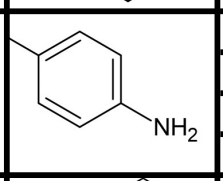	(*R*,*R'*)	1864.00 ± 248.00[a]	42.80 ± 11.3[d]	2.42 ± 0.58[c]	122.84 ± 17.8[c]	6.50 ± 0.95[e]			0.191 (0.095 - 0.361)	67.67 (60.80 - 74.84)
				(*R*,*S'*)	6040.00 ± 430.00[a]	187.00 ± 42.60[d]	15.59 ± 4.56[c]	170.88 ± 55.41[c]					
	NH_2Fen[d]			(*S*,*R'*)	30800.00 ± 3300.00[a]	463.00 ± 103.00[d]							
	AminoFen[b,d]			(*S*,*S'*)	28800.00 ± 1800.00[a]							403.645 (231.739 - 695.024)	87.55 (75.36 - 100.00)
5[a]	1-naphtylfenoterol	-CH_3	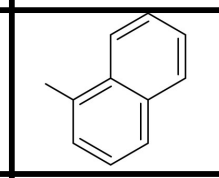	(*R*,*R'*)	241.44 ± 37.93[a]	3.66 ± 0.42[d]	12.50 ± 3.50[c]	129.20 ± 23.67[c]	1.57 ± 0.34[e]			0.079 (0.039 - 0.155)	58.52 (52.36 - 65.25)
				(*R*,*S'*)	341.39 ± 32.06[a]	3.67 ± 1.32[d]	2.70 ± 0.84[c]	112.10 ± 42.45[c]	1.19 ± 0.38[e]				
	1-NaphtylFen[d]			(*S*,*R'*)	1783.78 ± 208.88[a]	57.60 ± 4.73[d]	66.70 ± 32.13[c]	141.60 ± 3.70[c]	14.87 ± 5.59[e]				
	1-NapFen[b,d]			(*S*,*S'*)	2535.08 ± 295.08[a]	615.00 ± 85.20[d]	29.70 ± 10.57[c]	111.40 ± 24.25[c]	229.17 ± 57.40[e]				
6[a]	not assigned	-CH_3	-CH_2-CH_2-CH_3	(*R*,*R'*)	9280.00 ± 900.00[a]								
				(*R*,*S'*)	31400.00 ± 1700.00[a]								
				(*S*,*R'*)	>100000.00[a]								
				(*S*,*S'*)	56420.00 ± 5190.00[a]								
7[a]	desmethylphenylfenoterol	-H	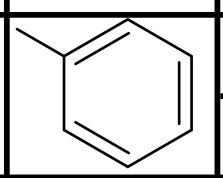	(*R*)	10500.00 ± 1500.00[a]								
				(*S*)	20600.00 ± 3700.00[a]								
52[c]	ethylfenoterol	-CH_2-CH_3	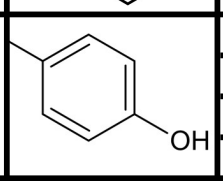	(*R*,*R'*)	1273.38 ± 81.11[c]	39.1 ± 5.38[d]	2.80 ± 0.90[c]	125.40 ± 5.74[c]	1.44 ± 0.27[e]			0.436 (0.283 - 0.664)	66.08 (61.69 - 70.51)
				(*R*,*S'*)	5758.67 ± 833.67[c]	294.00 ± 45.10[d]	16.60 ± 4.90[c]	98.00 ± 4.10[c]	17.88 ± 4.56[e]				
	EtFen[b,d]			(*S*,*R'*)	8317.64[c]*								
	EthylFen[d]			(*S*,*S'*)	22387.21[c]*								
53[c]	2-naphtylfenoterol	-CH_3	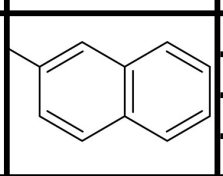	(*R*,*R'*)	404.60 ± 97.34[c]	4.52 ± 1.14[d]	0.40 ± 0.12[c]	90.30 ± 19.81[c]	1.91 ± 0.57[e]			0.100 (0.055 - 0.170)	67.51 (61.77 - 73.53)
				(*R*,*S'*)	509.8 ± 5.31[c]	134.00 ± 10.70[d]	7.60 ± 2.76[c]	88.70 ± 11.93[c]	72.6 ± 29.31[e]				
	2-NaphtylFen[d]			(*S*,*R'*)	6309.57[c]*								
	2-NapFen[b,d]			(*S*,*S'*)	6309.57[c]*								
54[c]	4′-methoxy-1-naphtylfenoterol	-CH_3	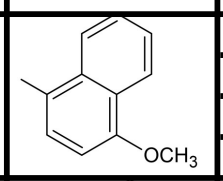	(*R*,*R'*)	277.88 ± 10.87[c]	13.30 ± 2.72[d]	3.90 ± 1.80[c]	105.90 ± 11.06[c]	3.98 ± 0.28[e]	-7.71 ± 0.08[g]	-8.76 ± 0.17[g]	0.118 (0.091 - 0.163)	65.05 (60.94 - 69.24)
				(*R*,*S'*)	317.85 ± 5.79[c]	12.70 ± 1.83[d]	4.00 ± 1.29[c]	118.20 ± 30.22[c]	4.37 ± 0.70[e]	-6.77 ± 0.14[g]	-7.52 ± 0.20[g]	4.853 (2.812 - 8.690)	74.82 (66.85 - 83.09)
	MNFen[b,d]			(*S*,*R'*)	575.44[c]*					-6.89 ± 0.09[g]	-7.77 ± 0.25[g]	0.773 (0.449 - 1.233)	70.49 (65.33 - 75.76)
	MNF[d]			(*S*,*S'*)	1230.27[c]*					-6.16 ± 0.14[g]	-7.08 ± 0.27[g]	93.972 (62.517 - 146.893)	75.31 (68.86 - 83.43)
55[c]	4′-hydroxy-1-naphtylfenoterol<br>HNF	-CH_3	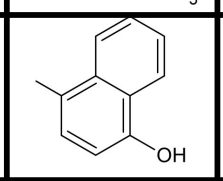	(*R*,*R'*)	162.18[c]*							0.025 (0.011 - 0.049)	53.06 (48.09 - 58.23)
56[c]	4′-fluoro-1-naphtylfenoterol	-CH_3	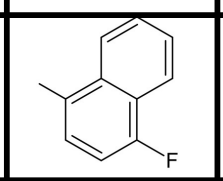	(*R*,*R'*)	173.78[c]*								
57[c]	4′-chloro-1-naphtylfenoterol	-CH_3	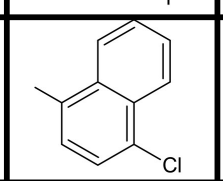	(*R*,*R'*)	120.23[c]*								
58[c]	4′-bromo-1-naphtylfenoterol	-CH_3	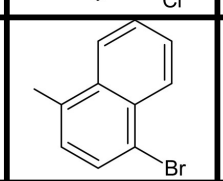	(*R*,*R'*)	114.82[c]*								
59[c]	4′-trifluoromethyl-1-naphtylfenoterol	-CH_3	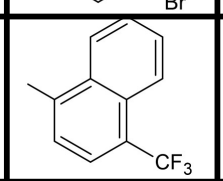	(*R*,*R'*)	112.20[c]*								
60[c]	4′-amino-1-naphtylfenoterol	-CH_3	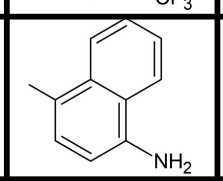	(*R*,*R'*)	1621.81[c]*		2.42 ± 0.58	122.84 ± 17.8				0.132 (0.071 - 0.211)	61.74 (56.21 - 67.36)
	ANF			(*R*,*S'*)			15.59 ± 4.56	170.88 ± 55.41					
61[c]	4′-methyl-1-naphtylfenoterol	-CH_3	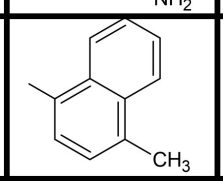	(*R*,*R'*)	389.05[c]*								
62[c]	4′-mercapto-1-naphtylfenoterol	-CH_3	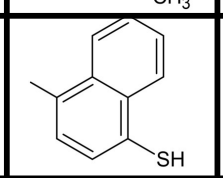	(*R*,*R'*)	416.87[c]*								
63[c]	4′-methylthio-1-naphtylfenoterol	-CH_3	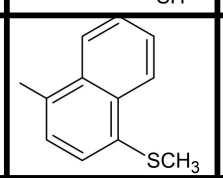	(*R*,*R'*)	398.11[c]*								
64[f]	4′-methoxy-ethylfenoterol	-CH_2-CH_3	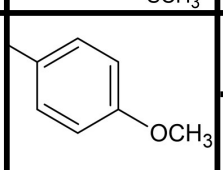	(*R*,*R'*)	420.00 ± 120.00[f]	35.7 ± 4.30[f]	1.63 ± 0.51[f]	156.00 ± 33.00[f]	14.00 ± 2.73[e]			0.167 (0.078 - 0.357)	47.55 (41.57 - 53.61)
				(*R*,*S'*)	20100.00 ± 4950.00[f]	4300.00 ± 1000.00[f]	1010.00 ± 240.00[f]	145.70 ± 8.80[f]					
65[f]	4′-methoxy-i-propylfenoterol	-$CH(CH_3)_2$	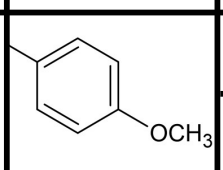	(*R*,*R'*)	>100000.00[f]	29200.00 ± 4400[f]	26200.00 ± 6500.00[f]	103.50 ± 7.80[f]				3318.945 (857.038 - 10327.614)	72.99 (44.68 - 100.00)
				(*R*,*S'*)	>100000.00[f]	3420.00 ± 320.00[f]	6100.00 ± 1500.00[f]	207.00 ± 56.00[l]	613.20 ± 199.10[e]				
66[f]	4′-methoxy-n-propylfenoterol	-CH_2-CH_2-CH_3	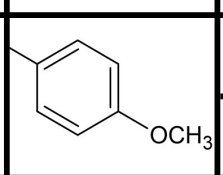	(*R*,*R'*)	7600.00 ± 230.00[f]	250.00 ± 41.00[f]	462.00 ± 161.00[f]	147.20 ± 2.70[f]				15.205 (5.346 - 53.333)	70.62 (60.96 - 86.05)
				(*R*,*S'*)	>100000.00[f]	4740.00 ± 700.00[f]	6100.00 ± 1500.00[f]	197.00 ± 32.00[f]					
67[f]	4′-methoxy-desmethylfenoterol	-H	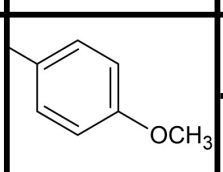	(*R*)	2670.00 ± 240.00[f]	101.00 ± 33.20[f]	10.19 ± 1.79[f]	114.20 ± 8.40[f]				3.170 (1.837 - 4.977)	50.81 (45.4 - 56.35)
				(*S*)	>100000.00[f]	1150.00 ± 410.00[f]	210.00 ± 96.00[f]	131.00 ± 43.00[f]					
70	4′-methoxy-1-naphtyl-ethylfenoterol	-CH_2-CH_3	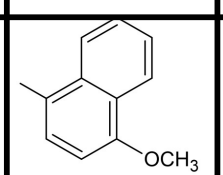	(*R*,*R'*)								0.423 (0.323 - 0.562)	58.83 (55.82 - 61.89)
71	1-naphtyl-ethylfenoterol	-CH_2-CH_3	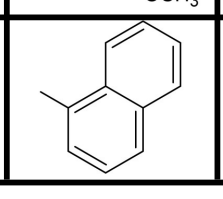	(*R*,*R'*)								0.056 (0.043 - 0.076)	59.88 (57.36 - 62.41)

† Binding affinities to the β_2AR determined using membranes obtained from HEK293 cells stably transfected with a plasmid DNA encoding human β_2AR (HEK293-β_2AR). Either [^{3}H]CGP-12177 or [^{3}H]MF was used as a marker ligand. Values labeled with an asterisk (*) were not determined experimentally but originated from a comparative molecular field analysis (CoMFA) model. Modeled values were provided only when no experimental data was available.

‡ Activity in cAMP accumulation assay in HEK293-β_2AR cells. Maximal cAMP accumulation was calculated in relation to isoproterenol (100%).

§ Inhibition of mitogenesis in 1231N1 astrocytoma cells as measured in [^{3}H]thymidine incorporation assay.

‖ ERK1/2 inhibition was assessed by measuring expression levels of the phosphoactive form of the kinases by the means of western blotting as described in the Materials and Methods section of this study. Maximal ERK1/2 inhibition was calculated in relation to DMSO-treated cells.

$ Activity in steady-state GTPase assay using membranes from Sf9 insect cells expressing β_2AR-$G_s\alpha$ fusion proteins. Values represent the potency [log(EC50)] to activate GTP hydrolysis.

€ Activity in adenylyl cyclase assay using membranes from Sf9 insect cells expressing β_2AR-$G_s\alpha$ proteins. Values represent the potency [log(EC50)] to stimulate cAMP production.

[a] Derived from Jozwiak et al. (2007).

[b] Derived from Woo et al. (2014).

[c] Derived from Jozwiak et al. (2010).

[d] Derived from Toll et al. (2012).

[e] Derived from Toll et al. (2011).

[f] Derived from Plazinska et al. (2014).

[g] Derived from Reinartz et al. (2015).

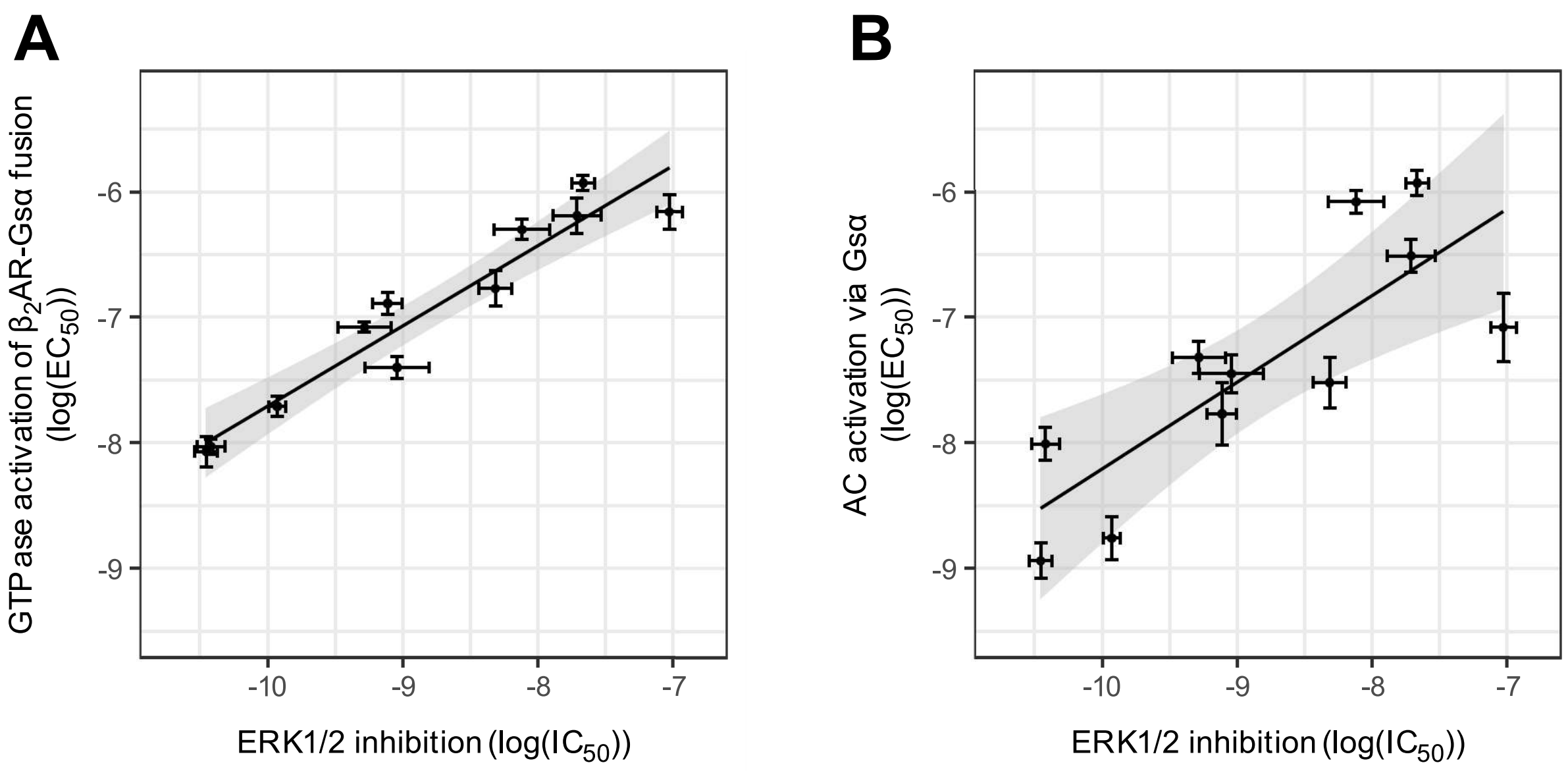

Figure S1. Correlation between ERK1/2 inhibition potency and canonical G_s-protein signaling efficacy. The potency of fenoterol stereoisomers and derivatives to inhibit ERK1/2 phosphorylation in UACC-647 cells [expressed as $\log(IC_{50})$] was correlated with their potency to activate canonical G_s-signaling pathways [expressed as $\log(EC_{50})$]. (A) Correlation with $G_s\alpha$ GTPase activity (r = 0.96). (B) Correlation with adenylyl cyclase (AC) activation (r = 0.82). GTPase and AC activation data were obtained from Reinartz et al. (2015). The plots include data for the stereoisomers of fenoterol, 4′-methoxyfenoterol (MF), and 4′-methoxy-1-naphthylfenoterol (MNF).

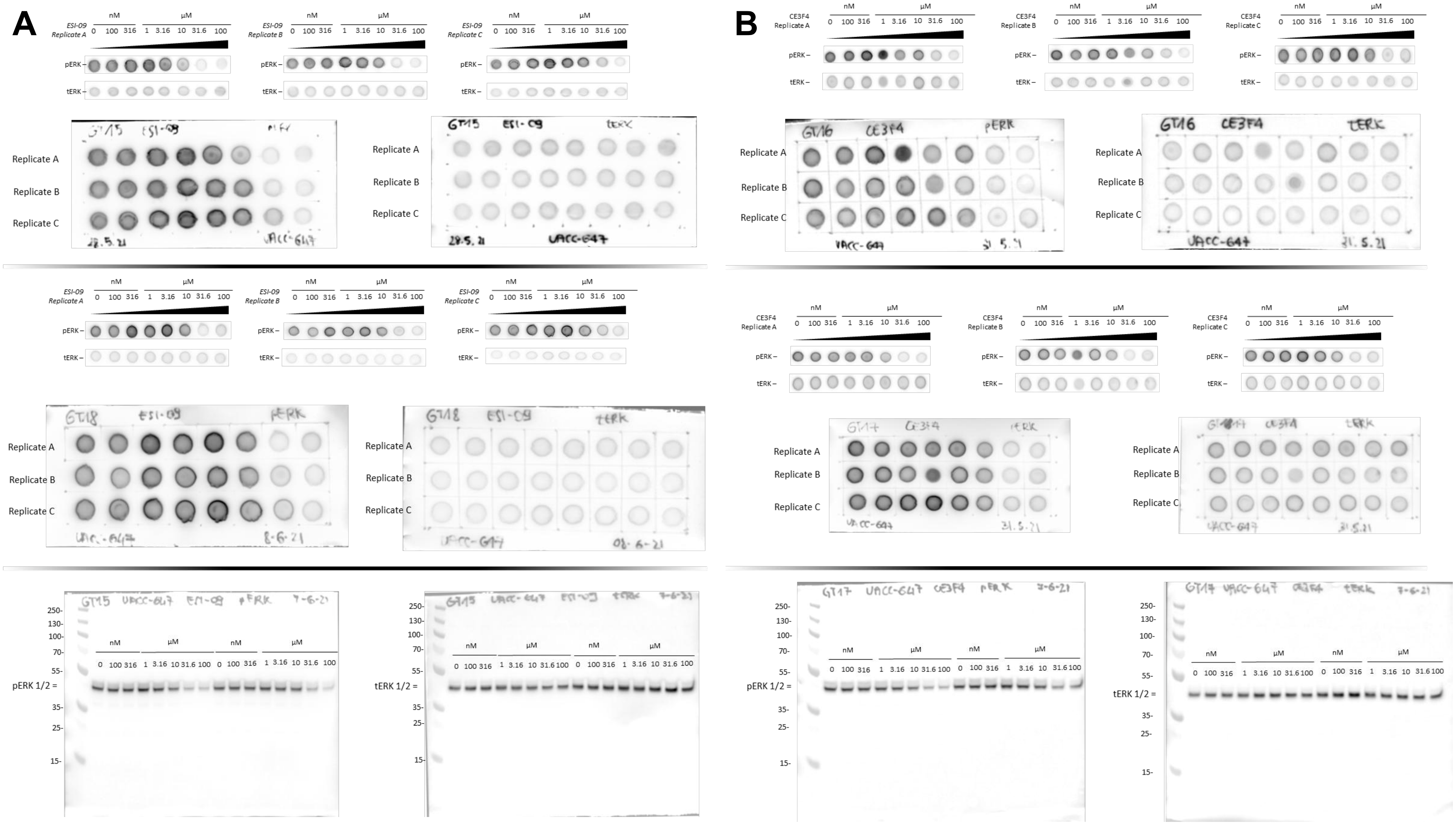

Figure S2. Uncropped blot images for Figure 4A (A) and Figure 4B (B).

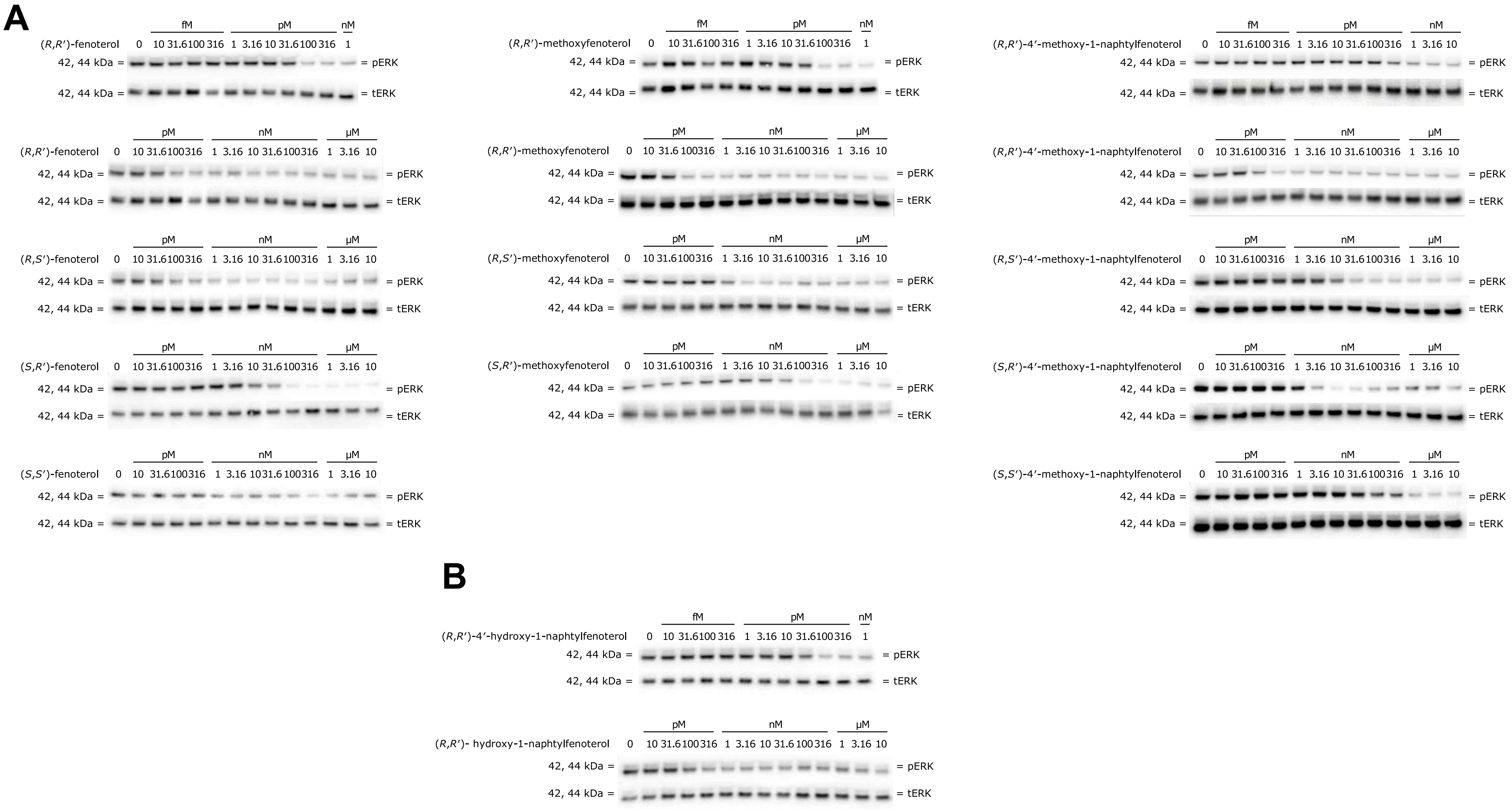

Figure S3. Representative western blot images for Figures 3A (A) and 3C (B). The compounds of interest were evaluated in half-log serial dilutions. The concentrations ranged from 10 pM to 10 µM for (*R*,*S′*), (*S*,*R′*), and (*S*,*S′*) isomers. (*R*,*S′*) isomers were assessed in two partially overlapping gradients: from 10 nM to 1 nM and from 10 pM to 10 µM.

References

Jozwiak, K., Khalid, C., Tanga, M. J., Berzetei-Gurske, I., Jimenez, L., Kozocas, J. A., Woo, A., Zhu, W., Xiao, R.-P., Abernethy, D. R., & Wainer, I. W. (2007). Comparative Molecular Field Analysis of the Binding of the Stereoisomers of Fenoterol and Fenoterol Derivatives to the β_2 Adrenergic Receptor. *Journal of Medicinal Chemistry*, *50*(12), 2903–2915. https://doi.org/10.1021/jm070030d

Jozwiak, K., Woo, A. Y.-H., Tanga, M. J., Toll, L., Jimenez, L., Kozocas, J. A., Plazinska, A., Xiao, R.-P., & Wainer, I. W. (2010). Comparative molecular field analysis of fenoterol derivatives: A platform towards highly selective and effective β2-adrenergic receptor agonists. *Bioorganic & Medicinal Chemistry*, *18*(2), 728–736. https://doi.org/10.1016/j.bmc.2009.11.062

Plazinska, A., Pajak, K., Rutkowska, E., Jimenez, L., Kozocas, J., Koolpe, G., Tanga, M., Toll, L., Wainer, I. W., & Jozwiak, K. (2014). Comparative molecular field analysis of fenoterol derivatives interacting with an agonist-stabilized form of the β2-adrenergic receptor. *Bioorganic & Medicinal Chemistry*, *22*(1), 234–246. https://doi.org/10.1016/j.bmc.2013.11.030

Reinartz, M. T., Kälble, S., Littmann, T., Ozawa, T., Dove, S., Kaever, V., Wainer, I. W., & Seifert, R. (2015). Structure-bias relationships for fenoterol stereoisomers in six molecular and cellular assays at the β2-adrenoceptor. *Naunyn-Schmiedeberg's Archives of Pharmacology*, *388*(1), 51–65. https://doi.org/10.1007/s00210-014-1054-5

Toll, L., Jimenez, L., Waleh, N., Jozwiak, K., Woo, A. Y. H., Xiao, R. P., Bernier, M., & Wainer, I. W. (2011). β2-adrenergic receptor agonists inhibit the proliferation of 1321N1 astrocytoma cells. *Journal of Pharmacology and Experimental Therapeutics*, *336*(2), 524–532. https://doi.org/10.1124/jpet.110.173971

Toll, L., Pajak, K., Plazinska, A., Jozwiak, K., Jimenez, L., Kozocas, J. A., Tanga, M. J., Bupp, J. E., & Wainer, I. W. (2012). Thermodynamics and docking of agonists to the β(2)-adrenoceptor determined using [(3)H](R,R′)-4-methoxyfenoterol as the marker ligand. *Molecular Pharmacology*, *81*(6), 846–854. https://doi.org/10.1124/mol.111.077347

Woo, A. Y. H., Jozwiak, K., Toll, L., Tanga, M. J., Kozocas, J. A., Jimenez, L., Huang, Y., Song, Y., Plazinska, A., Pajak, K., Paul, R. K., Bernier, M., Wainer, I. W., & Xiao, R. P. (2014). Tyrosine 308 is necessary for ligand-directed Gs protein-biased signaling of β2-adrenoceptor. *Journal of Biological Chemistry*, *289*(28), 19351–19363. https://doi.org/10.1074/jbc.M114.558882
